# U.S. Preparedness and Response to Increasing Clade I Mpox Cases in the Democratic Republic of the Congo — United States, 2024

**DOI:** 10.15585/mmwr.mm7319a3

**Published:** 2024-05-16

**Authors:** Jennifer H. McQuiston, Richard Luce, Dieudonne Mwamba Kazadi, Christian Ngandu Bwangandu, Placide Mbala-Kingebeni, Mark Anderson, Joanna M. Prasher, Ian T. Williams, Amelia Phan, Victoria Shelus, Anna Bratcher, Gnakub Norbert Soke, Peter N. Fonjungo, Joelle Kabamba, Andrea M. McCollum, Robert Perry, Agam K. Rao, Jeff Doty, Bryan Christensen, James A. Fuller, Nicolle Baird, Jasmine Chaitram, Christopher K. Brown, Amy E. Kirby, David Fitter, Jennifer M. Folster, Mushtaq Dualeh, Regan Hartman, Stephen M. Bart, Christine M. Hughes, Yoshinori Nakazawa, Emily Sims, CDC 2024 Clade I Mpox Response Team, Athalia Christie, Christina L. Hutson

**Affiliations:** ^1^Division of High Consequence Pathogens and Pathology, National Center for Emerging and Zoonotic Infectious Diseases, CDC; ^2^Division of Global Health Protection, Global Health Center, CDC; ^3^National Public Health Institute, Kinshasa, Democratic Republic of the Congo; L’Institut National de Recherche Biomédicale, Kinshasa, Democratic Republic of the Congo; ^5^Office of the Director, Office of Readiness and Response, CDC; ^6^Division of Readiness and Response Science, Office of Readiness and Response, CDC; ^7^Office of Public Health Data, Surveillance, and Technology, CDC; ^8^Division of Global HIV and TB, Global Health Center, CDC; ^9^Influenza Division, National Center for Immunization and Respiratory Diseases, CDC; ^10^Global Immunization Division, Global Health Center, CDC; ^11^Office of the Director, Center for Laboratory Systems and Response, CDC; ^12^Division of Emergency Operations, Office of Readiness and Response, CDC; ^13^Division of Infectious Disease Readiness and Response, National Center for Emerging and Zoonotic Infectious Diseases, CDC; ^14^Division of Global Migration Health, National Center for Emerging and Zoonotic Infectious Diseases, CDC; ^15^Division of Core Laboratory Services and Response, National Center for Emerging and Zoonotic Infectious Diseases, CDC; ^16^Office of the Director, National Center for Emerging and Zoonotic Infectious Diseases, CDC; ^17^Office of the Director, Global Health Center, CDC.

**Summary**

**What is already known about this topic?**

Compared with clade II monkeypox virus (MPXV), which caused the 2022 global mpox outbreak, clade I MPXV can result in more persons with severe illness and higher mortality.

**What is added by this report?**

The increasing number of reported suspected clade I mpox cases in the Democratic Republic of the Congo (DRC) poses a global threat for potential spread. No clade I cases have been reported in countries without endemic transmission. CDC is supporting DRC’s response and containment efforts and ensuring U.S. preparedness by increasing awareness and surveillance, expanding clade I diagnostic testing capacity, and communicating guidance.

**What are the implications for public health practice?**

U.S. clinicians and public health practitioners should be alert for possible cases in travelers from DRC and request clade-specific testing. Appropriate medical treatment is critical given the potential for severe illness, and contact tracing and containment strategies, including isolation, behavior modification and vaccination, will be important to prevent spread if any U.S. clade I mpox cases occur.

## Abstract

Clade I monkeypox virus (MPXV), which can cause severe illness in more people than clade II MPXVs, is endemic in the Democratic Republic of the Congo (DRC), but the country has experienced an increase in suspected cases during 2023–2024. In light of the 2022 global outbreak of clade II mpox, the increase in suspected clade I cases in DRC raises concerns that the virus could spread to other countries and underscores the importance of coordinated, urgent global action to support DRC’s efforts to contain the virus. To date, no cases of clade I mpox have been detected outside of countries in Central Africa where the virus is endemic. CDC and other partners are working to support DRC’s response. In addition, CDC is enhancing U.S. preparedness by raising awareness, strengthening surveillance, expanding diagnostic testing capacity for clade I MPXV, ensuring appropriate specimen handling and waste management, emphasizing the importance of appropriate medical treatment, and communicating guidance on the recommended contact tracing, containment, behavior modification, and vaccination strategies.

## Introduction

The global clade II monkeypox virus (MPXV) outbreak that began in 2022 demonstrated the pandemic potential of mpox (*1*). Clade I MPXV is endemic in several Central African countries, including the Democratic Republic of the Congo (DRC); clade I is generally associated with higher case fatality rates (CFRs) (1.4% to >10%) compared with clade II MPXV (0.1% to 3.6%) (*1*–*3*). MPXV can spread to persons from contact with infected wildlife, or through close, prolonged contact with persons infected with MPXV; the global clade II MPXV outbreak spread primarily via sexual contact among gay, bisexual, or other men who have sex with men (MSM) (*1*). During 2023–2024, DRC has reported an unprecedented number of suspected clade I MPXV infections. Neighboring countries and the global community should help support DRC’s effort to contain the virus as well as prepare for the possibility of further spread. This report describes investigation of cases in DRC, CDC’s support to DRC, and U.S. public health preparedness activities to date.

## DRC Investigation and Findings

### Epidemiology of Clade I MPXV in DRC

Data from DRC’s national infectious disease surveillance system were analyzed for this report. This activity was reviewed by CDC, deemed not research, and was conducted consistent with applicable federal law and CDC policy.^†^

**FIGURE 1:**
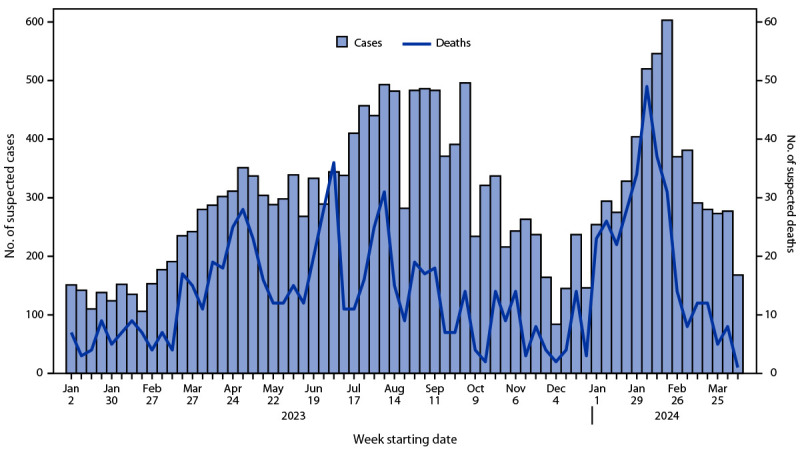
Suspected clade I mpox cases and deaths* — Democratic Republic of the Congo, January 1, 2023–April 14, 2024 * Reporting of data from multiple sources might result in minor discrepancies between the numbers of cumulative cases and deaths reported to CDC by the Democratic Republic of the Congo Ministry of Health and those that appear in the figure.

**FIGURE 2:**
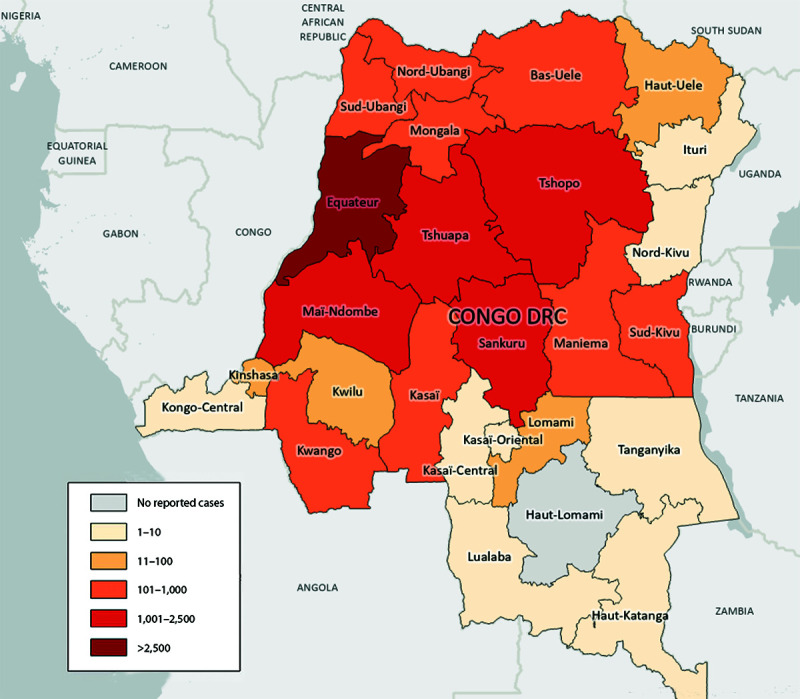
Number of suspected clade I mpox cases, by province — Democratic Republic of the Congo, January 1, 2023–April 14, 2024

**FIGURE 3:**
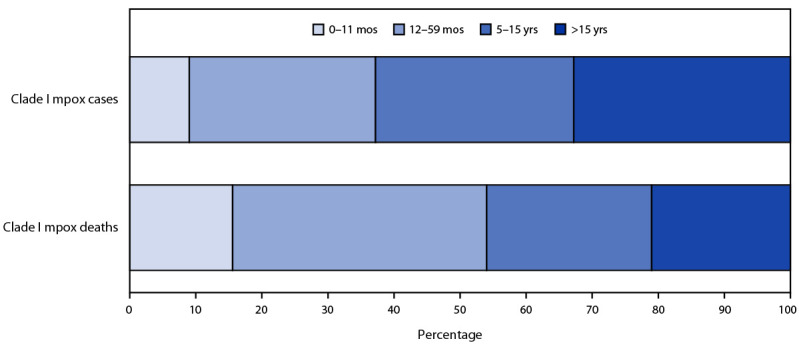
Age distribution of suspected clade I mpox cases and deaths — Democratic Republic of the Congo, January 1, 2023–April 14, 2024

During January 1, 2023–April 14, 2024, DRC reported multiple provincial-level outbreaks, comprising 19,919 cases of suspected clade I mpox^§^ and 975 (4.9%) deaths (Figure 1). During 2023 and 2024, clade I mpox cases were reported from 25 of 26 provinces and, for the first time, from the capital city of Kinshasa (Figure 2). In two outbreaks, sexual transmission of clade I MPXV was reported among MSM and both male and female sex workers and their contacts^¶^ (*4*). Overall, two thirds (67%) of suspected cases and more than three quarters (78%) of suspected deaths have occurred in persons aged ≤15 years; children aged 12–59 months accounted for 28% of all suspected cases (Figure 3). Demographic characteristics of patients differed among provinces, with suspected cases in some provinces (e.g., Equateur) occurring primarily in persons aged ≤15 years (69%), whereas in other provinces (e.g., South Kivu and Kinshasa) persons aged >15 years accounted for the largest proportion of cases (69%). During January 1, 2023–April 14, 2024, 50% of DRC’s suspected mpox cases were reported from Equateur province; during this period, Equateur province also reported an elevated CFR (5.7%) compared with CFRs reported elsewhere in the country (4.3%). In remote locations, many suspected cases are not tested for MPXV, and testing varies widely by province. Among 2,016 specimens tested during January 1, 2023–March 24, 2024 (accounting for 11% of all suspected cases), 1,302 (65%) were laboratory-confirmed as positive. In a subset of 480 laboratory-confirmed positive cases reported during January 1–March 24, 2024, for which patient age was reported, 239 (50%) occurred in persons aged ≤15 years.

### Mpox Transmission and Outbreaks in DRC

Limited data on genetic diversity among circulating clade I MPXV strains (L’Institut National de Recherche Biomédicale, DRC, unpublished data, 2024) suggests that outbreaks involve multiple introductions from animal hosts within DRC, rather than a single introduction that has spread nationwide. However, communicable spread to close contacts within households likely contributes substantially to the case count. Geographic differences in demographic characteristics and viral genetic diversity suggest various transmission drivers (i.e., zoonotic, household, or sexual) in different provinces, resulting in a complex epidemiologic picture. In South Kivu province, where sexual transmission has been reported, a distinct lineage of clade I MPXV was observed, including genetic markers suggesting sustained human-to-human transmission and a deletion in a nonessential segment of the genome that includes the C3 gene targeted by CDC’s clade I–specific polymerase chain reaction (PCR) test (*5*,*6*).

### CDC Support to DRC

CDC’s support for DRC’s mpox-related activities during the last 15 years has included establishing laboratory testing and training, supporting diagnostic testing and genetic sequencing, conducting JYNNEOS vaccine clinical research, and training frontline health care workers. In response to the current outbreak, the U.S. government established an interagency response team to coordinate support to DRC and neighboring countries as well as to direct U.S. preparedness efforts. The U.S. government is providing funding, technical assistance, and personnel deployments to support the DRC response.

## U.S. Public Health Preparedness and Response

### Notifications and At-Risk U.S. Populations

CDC issued a Health Alert Network notice on December 7, 2023, urging U.S. clinicians to consider clade I MPXV infection in persons with mpox signs and symptoms who had recently been in DRC. The notice recommended expedited clade-specific testing for those patients.**

CDC also issued a Level 2 Travel Health Notice for DRC.^††^ To date, no cases of clade I mpox have been reported in the United States or in any countries where the virus is not endemic. However, given the documented sexual transmission of clade I MPXV in DRC, persons engaging in certain sexual behaviors (e.g., MSM with multiple sexual partners and sex workers) might be at increased risk if clade I mpox is introduced into the United States. Although clade I MPXV transmission in DRC is more commonly reported among children, widespread transmission among children in the United States is considered much less likely because of 1) absence of zoonotic reservoirs, 2) fewer household occupants, and 3) widely available cleaning and hygiene resources.

### Current CDC Activities and Recommendations

Working with U.S. jurisdictional partners, CDC revised existing mpox case reporting forms to include clade I MPXV–specific laboratory results to facilitate delivery of timely situational awareness to decision-makers. After technical consultation with CDC, the U.S. Department of Transportation published updated guidance,^§§^ stating that diagnostic specimens (other than clade I MPXV culture) and clinical waste containing either clade I or clade II MPXV should be handled as Category B infectious substances,^¶¶^ a classification for infectious substances that generally are not capable of causing permanent disability or life-threatening or fatal disease in otherwise healthy persons or animals. A March 2024 Internet survey*** of 100 U.S. health care providers (CDC, unpublished data, 2024) demonstrated gaps in knowledge about the diagnosis and treatment of clade I mpox; approximately two thirds of providers wanted more clinical management guidance. CDC has updated its online content to provide clade I testing and reporting information^†††^ and has shared updates on testing and preparedness with health care providers and state and local health departments. U.S. guidelines for clinical mpox management are applicable for clade I MPXV, with patient management decisions based on clinical presentation, disease severity, and underlying health risks, rather than virus clade (*7*). Diagnosis and reporting of clade I MPXV infections to health officials is important for limiting onward transmission by aiding early containment measures, including contact tracing, isolating patients, offering JYNNEOS vaccine to contacts, and strictly adhering to recommended infection prevention and control practices in health care settings.^§§§^ In the United States, only 23% of persons at risk for clade II MPXV infection have completed the 2-dose JYNNEOS vaccination series.^¶¶¶^ Through ongoing work with advocates and partners, CDC encourages persons who currently are at risk for clade II MPXV infection to be vaccinated with 2 doses of JYNNEOS.**** JYNNEOS is included in the routine adult immunization schedule and became available commercially in the United States in April, 2024.^††††^ An additional benefit of vaccination is protection against clade I MPXV infection.

### MPXV Testing

The U.S. mpox diagnostic testing strategy includes CDC’s Food and Drug Administration (FDA)–cleared nonvariola orthopoxvirus (NVO) PCR test. A positive test result provides a presumptive diagnosis of mpox; however, the test cannot distinguish between clades (*8*). In addition, some Laboratory Response Network, state public health, and commercial laboratories offer laboratory-developed tests (LDTs)^§§§§^ and FDA emergency use–authorized tests that can differentially detect clade II (thereby ruling out clade I); a few offer LDTs that differentially detect clade I. However, because of concerns about potential genomic deletions affecting test efficacy (*5*,*6*), CDC recommends that the NVO test be used in addition to clade-specific testing, and that positive NVO or negative clade II test results be further investigated through sequence analysis.^¶¶¶¶^ In addition, CDC conducts surveillance using clade-specific PCR testing and sequence analysis of NVO-positive specimens received from other U.S. laboratories. During December 1, 2023–April 14, 2024, among 343 NVO-positive specimens tested by CDC, no clade I MPXV infections were identified. Similarly, no clade I MPXV was confirmed among approximately 900 specimens tested during this period by other U.S. laboratories with the capacity to detect clade I MPXV or to predict a high likelihoood of clade I MPXV (NVO-positive and clade II MPXV–negative). U.S. wastewater surveillance testing for mpox***** was enhanced in late 2023 with a recommendation to use the NVO test; as of April 22, 2024, a total of 186 sites in 32 jurisdictions are using tests that can detect both clades. To date, all positive detections have coincided with locations with known clade II MPXV occurrence. During December 22, 2023–April 11, 2024, a total of 282 samples were collected from four U.S. airports and analyzed using the NVO test; no MPXV-positive samples were found.

## Discussion

Ten years ago, the 2014 West Africa Ebolavirus outbreak demonstrated the risks associated with a delayed global response to a serious pandemic threat (*9*). During the 2022 clade II MPXV outbreak, the United States launched a robust domestic response based on 2 decades of smallpox preparedness; however, the global public health community missed earlier opportunities to recognize the threat and help contain clade II mpox, which was spreading person-to-person in Nigeria as early as 2016 (*10*). The recent increases in clade I MPXV transmission in DRC pose a new risk for global spread if the virus is not urgently contained. Reports of increased mpox in some bordering countries with endemic MPXV, including 19 confirmed cases in Republic of the Congo, reinforce this concern.^†††††^ In the United States, clinicians and public health practitioners should be aware of clade I MPXV and request clade-specific testing for possible cases in travelers from DRC. In addition to preparing for the possibility of spread beyond DRC, support to DRC from global partners is needed as the country works to increase testing and surveillance for clade I MPXV. Although vaccines and therapeutics are not currently authorized for use in DRC, the National Immunization Technical Advisory Group in DRC recently released recommendations supporting their use as part of the country’s response. Collaboration among global public health partners is now urgently needed to assist DRC in procuring and delivering sufficient vaccine where it is most needed.

## Footnotes

* These senior authors contributed equally to this report.

† 45 C.F.R. part 46, 21 C.F.R. part 56; 42 U.S.C. 241(d); 5 U.S.C. Sect. 552a; 44 U.S.C. Sect. 3501 et seq.

§ In DRC, a “suspect case” is defined as “a person with sudden high fever followed by a vesiculo-pustular rash predominantly on the face and present on the palms of the hands and soles of the feet; or presence of at least 5 smallpox-type scars; or, anyone with fever >38.3°C (101°F), severe headache, lymphadenopathy, back pain, myalgia and intense asthenia, followed one to three days later by a progressive rash that often starts on the face and then spreads elsewhere on the body, including the soles of the feet and palms of the hands.”

¶
https://www.medrxiv.org/content/10.1101/2024.03.05.24303395v1

**
https://emergency.cdc.gov/han/2023/han00501.asp?trk=public_post_comment-text

††
https://wwwnc.cdc.gov/travel/notices/level2/monkeypox-democratic-republic-of-congo

§§
https://www.phmsa.dot.gov/transporting-infectious-substances/planning-guidance-handling-category-solid-waste

¶¶
https://www.phmsa.dot.gov/transporting-infectious-substances/transporting-infectious-substances-safely

***
https://styles.porternovelli.com/pn-view-panels/5

†††
https://www.cdc.gov/poxvirus/mpox/lab-personnel/report-results.html

§§§
https://www.cdc.gov/poxvirus/mpox/clinicians/infection-control-healthcare.html

¶¶¶
https://www.cdc.gov/poxvirus/mpox/cases-data/mpx-jynneos-vaccine-coverage.html

****
https://www.cdc.gov/poxvirus/mpox/interim-considerations/overview.html

††††
https://www.cdc.gov/vaccines/schedules/hcp/imz/adult.html

§§§§
https://www.fda.gov/medical-devices/in-vitro-diagnostics/laboratory-developed-tests

¶¶¶¶
https://www.cdc.gov/locs/2022/09-02-2022-lab-alert-MPXV_TNF_Receptor_Gene_Deletion_May_Lead_False_Negative_Results_Some_MPXV_Specific_LDTs.html

*****
https://www.cdc.gov/nwss/wastewater-surveillance/mpox-data.html

†††††
https://www.reuters.com/world/africa/congo-republic-declares-mpox-epidemic-2024-04-24/
